# Durable Left Ventricular Assist Device Outflow Graft Obstructions: Clinical Characteristics and Outcomes

**DOI:** 10.3390/jcm12062430

**Published:** 2023-03-22

**Authors:** Carli J. Peters, Robert S. Zhang, Mahesh K. Vidula, Jay Giri, Pavan Atluri, Michael A. Acker, Christian A. Bermúdez, Allison Levin, Kim Urgo, Joyce Wald, Jeremy A. Mazurek, Thomas C. Hanff, Lee R. Goldberg, Dinesh Jagasia, Edo Y. Birati

**Affiliations:** 1 Department of Medicine, Perelman School of Medicine, University of Pennsylvania, Philadelphia, PA 19104, USA; 2 Division of Cardiology, Perelman School of Medicine, University of Pennsylvania, Philadelphia, PA 19104, USA; 3 Division of Cardiovascular Medicine, NYU Langone Health, New York, NY 10016, USA; 4 Cardiovascular Outcomes, Quality, and Evaluative Research Center, University of Pennsylvania, Philadelphia, PA 19104, USA; 5 Cardiothoracic Surgery, Perelman School of Medicine, University of Pennsylvania, Philadelphia, PA 19104, USA; 6 Division of Cardiology, Duke University Medical Center, Durham, NC 27710, USA; 7 Division of Cardiovascular Medicine, University of Utah School of Medicine, Salt Lake City, UT 84132, USA; 8 The Lydia and Carol Kittner, Lea and Benjamin Davidai Cardiovascular Division, Tzafon (Poriya) Medical Center, Azrieli Faculty of Medicine, Bar-Ilan University, Ramat Gan 5290002, Israel

**Keywords:** LVAD, outflow graft, stenosis, stent

## Abstract

Purpose: We report on the clinical course and management of patients supported with durable implantable LVADs who developed outflow graft obstructions at a large academic center. Methods: We performed a retrospective review of patients receiving LVAD support from 2012 through 2020. Patients who developed an outflow graft obstruction diagnosed by computed tomography angiography (CTA) or angiogram were identified, and patient characteristics and outcomes were reported. Results: Of the 324 patients supported by LVAD at our institution, 11 patients (3.4%) were diagnosed with outflow graft obstructions. The most common presentation was low flow alarms, which was present in 10/11 patients, and the remaining patient presented with lightheadedness. Patients had minimal LDH elevation with 8/11 presenting with less than 2-fold the upper limit of normal. Transthoracic echocardiograms were not diagnostic, but CTA enabled non-invasive diagnoses in 8/11 of the patients. Three patients with extrinsic compression of the outflow graft successfully underwent endovascular stent placement, and three patients with outflow cannula kinks received supportive care. Of the five patients diagnosed with intraluminal thromboses, one received a heart transplant, one underwent an outflow graft revision, and three received supportive care due to comorbidities. Conclusion: Outflow graft obstructions remain a rare, but serious complication. The true prevalence of this entity is likely underestimated due to the non-specific clinical presentation. CTA is a pivotal non-invasive diagnostic step. Patients with external compression were successfully treated with endovascular stenting.

## 1. Introduction

Durable mechanical assist devices have emerged as an important therapeutic option for patients with advanced heart failure, both as a bridge to heart transplantation and as a destination therapy [1,2]. Despite recent improvements in survival with continuous flow left ventricular assist devices (LVADs), device-specific complications continue to affect patients’ outcomes. In the Multicenter Study of MagLev Technology in Patients Undergoing Mechanical Circulatory Support Therapy with HeartMate 3 (MOMENTUM 3), the HeartMate 3 centrifugal-flow left ventricular assist device reported significantly less pump thromboses at 1.4% compared to 13.9% for axial-flow LVADs [3]. While the rate of pump thromboses decreased, cases of outflow graft obstructions due to a specific twisting of the graft prompted an FDA recall [4]. In regard to outflow graft pathology, other kinks, intramural outflow graft thromboses, and external compressions have been reported in case reports and small case series [3,5,6,7,8,9,10,11]. Although the prevalence of this entity is likely higher than described, given the small number of published cases, there are limited recommendations on approaches to diagnosis and treatment. We present the clinical course and management of consecutive patients who were diagnosed with outflow graft obstruction over an 8-year period at a high-volume VAD center. We also highlight the importance of CTA as an important diagnostic step in assessing outflow graft obstructions. 

## 2. Materials and Methods

We performed a retrospective study among patient supported with continuous flow LVADs who developed outflow graft obstructions. Data were collected from electronic medical records of patients treated at the Hospital of the University of Pennsylvania between 2012 and 2020. Ambulatory and hospitalized patients with LVADs (HeartMate 2 (Abbott, Abbott Park, IL, USA), HeartMate 3 (Abbott, Abbott Park, IL, USA), and HeartWare (Medtronic Inc, Minneapolis, MN, USA)), and diagnoses of outflow graft obstructions were included. An outflow graft obstruction was defined per diagnosis on computed tomography angiography (CTA) or via direct visualization and confirmatory pressures on a cardiac angiogram.

Baseline characteristics and outcomes were obtained via a review of electronic medical records for each patient. Outcomes were described through the time of transplant, death, or September 2020, whichever occurred first. At our institution, we use aspirin and warfarin for patients with continuous flow LVADs with a goal INR of 2.0–3.0. This study was approved by the University of Pennsylvania Institutional Review Board. The requirement for specific informed consent for this study was waived on the basis of minimal privacy risk. De-identified data that support the findings of this study are available from the corresponding author upon request.

## 3. Results 

### 3.1. Study Population

We identified 324 patients implanted with LVADs at our institution between January 2012 and September 2020, of whom 11 (3.4%) were diagnosed with an outflow graft obstruction (Table 1). At the time of diagnosis, the mean (±SD) age in years was 54.8 (±8.7). Ten of the eleven patients were male, and nine had non-ischemic etiology of heart failure. The implantation strategy for all patients who had an outflow graft obstruction was via a full sternotomy with the outflow graft connected to the ascending aorta. HeartWare was implanted in 6 of the 11 patients, while 4 were implanted with HeartMate3, and 1 was implanted with HeartMate2. With regard to the goals of therapy, 8 of 11 patients were implanted as a destination therapy, and the remaining 3 were implanted as a bridge to transplant. At the time of diagnosis, 10 of 11 patients were being treated with warfarin and 8 patients were being treated with aspirin. 

### 3.2. Presentation

The median time to presentation with an outflow graft obstruction after LVAD implantation was 2.4 years (IQR 0.7, 3.4) (Table 2). The most common cause for presentation was low flow alarms, as was reported in 10 of 11 patients. Similar to the current recommendation, our institution sets the low flow alarm for a patient implanted with HeartWare when there is a decrease of >2 L/min below the patient-specific average values [12]. The default low flow limit for Heartmate2 and Heartmate3 is 2.5 L/min. Aside from low flow alarms, 5 of the 11 patients were otherwise asymptomatic. Of those who had symptoms (55%), the most common presentation was lightheadedness. Other non-specific symptoms included shortness of breath, dark urine, and fatigue. In regard to laboratory measurements, five patients had an acute kidney injury (AKI) with a median creatinine of 1.75 (IQR 0.95, 2.37) on presentation. AKI was defined as an increase in serum creatinine of 0.3 mg/dL or an increase in serum creatinine 1.5 times the baseline. Four patients had an increase in bilirubin of more than 1 mg/dL compared to the baseline, and all patients had relatively stable transaminases. Although 8 of 11 patients had an LDH higher than the normal limit of 192 units/L, the degree of elevation was low: 8 patients had an LDH less than 2-fold the normal limit and only 1 patient had an elevated LDH value at 5-fold the normal limit. The patient with a highly elevated LDH was being treated with aspirin and warfarin at the time of diagnosis (Table 1).

### 3.3. Diagnosis and Imaging

**Figure 1:**
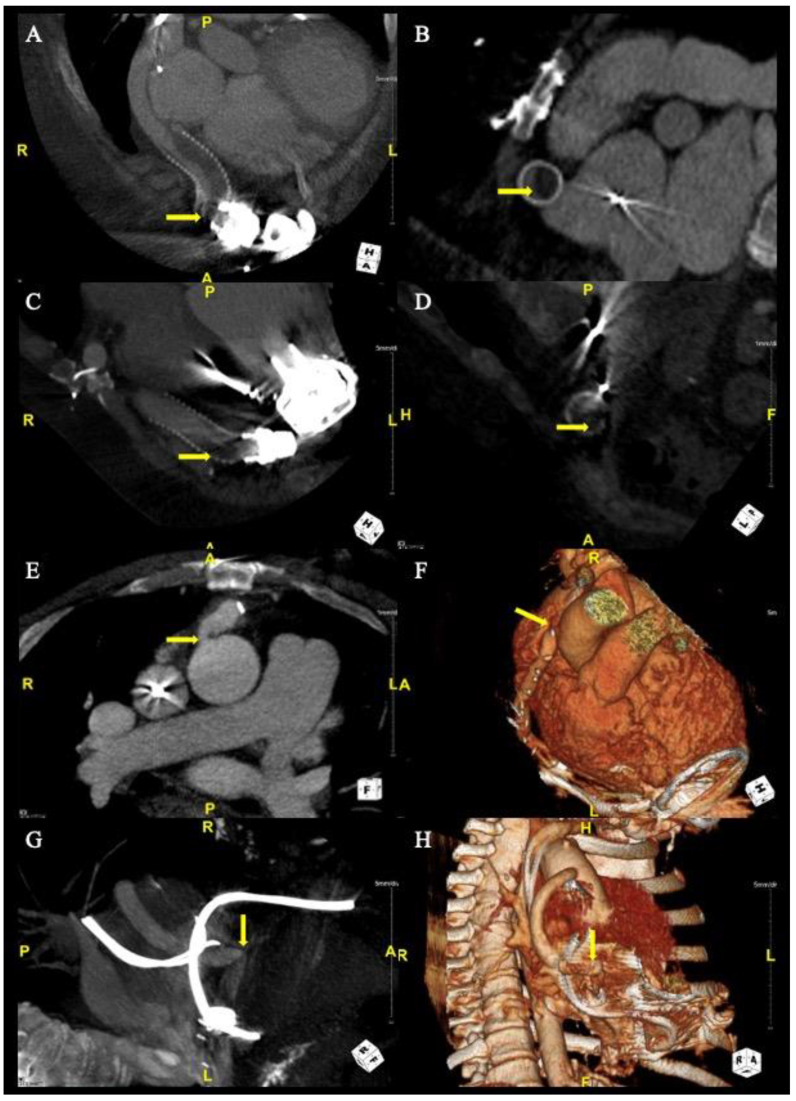
Computed Tomography Angiography of Obstructions. For case 1, CTA (Panel **A**) demonstrates stenosis with bend relief disconnect and CTA (Panel **B**) demonstrates a partially occlusive thrombus. For case 5, CTAs (Panel **C**,**D**) demonstrate partially occlusive thrombus. For case 8, CTAs (Panel **E**,**F**) demonstrate ascending aorta anastomotic stenosis. For case 11, CTAs (Panel **G**,**H**) demonstrate outflow cannula kink.

To investigate for an obstruction, 9 of the 11 patients underwent CTA (Table 3). Two patients did not have CTAs performed on presentation: one patient was already diagnosed with obstruction on angiogram prior to the CTA decision, and the other patient had a CTA withheld due to them presenting creatinine at twice their baseline. There is no creatinine limit prohibiting CTA at our institution, and the decision to perform a CTA is made on a case-by-case basis. Of the nine CTAs performed, eight of them had identified and localized the obstruction (Table 3, Figure 1). The patient who had inconclusive findings on the CTA underwent an angiogram which confirmed the diagnosis (Table 3). All patients underwent a TTE on admission (Table 3). None of the patients were diagnosed with an outflow graft obstruction based on direct visualization of the TTE. The results of the TTE were overall nonspecific, but the most common findings were a persistent severe LV dilation with a mean (±SD) of 6.9 cm (±1.2), worsening mitral regurgitation from prior TTE, and the AV opening at baseline speed with every beat. Of the nine patients who had right heart catheterizations, all cardiac indexes exceeded 2.0 with variable pulmonary capillary wedge pressures (PCWPs) with a range of 10–29 mmHg (Table 3). Ultimately, 5 of 11 patients were diagnosed with outflow graft intraluminal thrombi, 3 patients had cannula kinks, and 3 patients had outflow tract stenoses due to extraluminal compression (Table 3). In regard to the patients with extraluminal compression, two patients had stenoses at the anatomic site with fibrotic tissue built up near the anastomoses, causing extraluminal compression. The other had fibrotic tissue built up between the bend relief and the cannula.

### 3.4. Interventions and Outcomes

The patients received a variety of interventions (Table 4). Decisions on best management were made in a multi-disciplinary discussion with heart failure cardiology, interventional cardiology, and cardiothoracic surgery teams. The three patients who had outflow graft stenoses due to extraluminal compression were managed with endovascular balloon-expandable covered stents (Gore Medical, Flagstaff, AZ, USA). There were no complications with the intervention, and all three patients were living at the time of study. Of the patients with outflow cannula kinks, they were largely managed with supportive care with one patient having her INR goal increased to 2.5–3.0. Similarly, these patients were living at the time of study. Of the five patients who had outflow graft intraluminal thromboses, one patient underwent an outflow graft revision, and one patient underwent a transplant, with both of these patients alive at the time of study. The remaining three patients were poor surgical candidates and received supportive care with one patient undergoing LVAD deactivation with an Amplatzer Septal Occluder. Ultimately, these patients expired during admission soon after diagnosis. 

## 4. Discussion

The true prevalence of an outflow graft obstruction is unknown, and the data are limited regarding the optimal diagnosis and management of this life-threatening entity. We describe the clinical course and various treatments of patients diagnosed with an outflow graft obstruction at a large academic institution. Our main findings are: 1. Most patients were largely asymptomatic and presented with low flow alarms. 2. The best modality for diagnosis was CTA, which enabled non-invasive diagnoses in 82% of the patients, compared to TTE which was not diagnostic in any patients. 3. LDH was only minimally elevated on presentation with 72% of the patients with an LDH of <2-fold the normal level.

Despite significant improvements in survival with continuous flow left ventricular assist devices (LVADs), serious device-specific complications may be associated with unfavorable outcomes. While pump thrombosis appears less common for the HeartMate3 than for axillary flow LVADs, reports of outflow graft twisting eventually led the FDA to recall the device [3,4]. Furthermore, cases of kinks, stenoses, and intraluminal thromboses have been reported [3,5,6,7,8,9]. 

In our cohort, the clinical demographics and presentation of patients diagnosed with an obstruction were varied. While there have been increased reports in the HeartMate 3 population [3], all three FDA-approved LVAD types implanted at our institution were present. The majority of patients were on aspirin and warfarin at the time of diagnosis. Previous case series have demonstrated low flow alarms being the common presentation [5,6,7,8], which is consistent with 10 of 11 patients in our cohort. It is important to note low flow alarms may be the only presenting sign. The patients had very few other symptoms, and when present, were subtle and non-specific including lightheadedness, shortness of breath, and fatigue. Furthermore, of the patients who had catheterizations, there were variable PCWPs, and CIs were all greater than two, suggesting that clinical heart symptoms and shock may not be sensitive or specific.

Similarly, laboratory results tended to be non-specific. About half of the patients had sustained AKIs, suggesting some form of end-organ damage in the setting of obstruction. Only four patients had a bilirubin level of 1 mg/dL greater than baseline, and all patients had relatively similar transaminases to their baseline. Previous studies have demonstrated elevated LDH trends predicting rotor pump thromboses [13]. Uriel et al. reported that an LDH of >5-fold the normal level was 100% and 92% specific for a diagnosis of pump thrombosis [14]. Our group previously reported 82% sensitivity with a 5-fold LDH cut-off in their series [15]. Interestingly, in this series of patients with outflow graft abnormality, LDH levels were significantly elevated above five times the normal limit only in a single patient, while 72% had only a mild LDH elevation of less than 2-fold the normal level. This low-level LDH elevation is seen in most LVAD patients even without outflow graft abnormalities. Our results are consistent with the previous case series of outflow graft obstructions which had mildly elevated LDHs [6,7,8]. Given the nature of outflow graft obstructions including kinks and extraluminal compression, and that the outflow graft thromboses are not at the rotor level where shearing may occur, it is unsurprising that LDH is not elevated. Ultimately, lab values had no role in diagnosing or excluding obstruction.

As is standard at our institution, when patients present with low flows, they are evaluated for common causes of low flow alarms including right ventricular failure, hypovolemia, cardiac tamponade (at early post-operative stages), and arrhythmias. When these causes are excluded, an assessment for outflow graft obstruction is performed. Noninvasive approaches have previously included ultrasound-based and contrast-based imaging techniques [12,16]. Although previous studies have suggested the utility of ramp studies in patients with suspected VAD thrombosis, its role in the evaluation of outflow graft pathology is unknown [12]. In our cohort, a TTE at baseline speed was performed on every patient, with the most common finding of AV opening at baseline speed and worsening MR from prior TTEs. While these findings may be suggestive of possible obstruction, they are neither specific nor sensitive. Ultimately, a CTA was the diagnostic study for the majority of patients. Additionally, a CTA was frequently able to localize the lesion, which is an essential component in management planning. In patients who were unable to have a CTA performed, an angiogram with graft pressures supported the diagnosis of outflow graft obstruction.

With respect to treatment, patients were successfully managed with a variety of interventions based on the etiology of the obstruction. At our institution, endovascular stenting was performed on patients who had extraluminal compression of the outflow graft causing stenosis. There were no complications associated with the procedures, and all patients were living at the time of study. Several small case series have recently shown endovascular interventions to be feasible, safe, and effective for outflow graft obstructions, and our experience is consistent with these findings [10,17,18,19,20,21]. Patients who developed kinks in their outflow graft obstruction were largely treated with supportive care, and those patients were alive at the time of study. The patients with intraluminal thromboses were managed with more aggressive interventions, with one patient receiving a transplant and one patient undergoing an outflow graft revision. Both of these patients were living at the time of study. In the event that patients were not candidates for surgical interventions, they were managed with supportive care. In our cohort, these three patients expired shortly after the diagnosis. Importantly, while the course for each obstruction type ultimately varied in severity and required different interventions, the original presentation was not suggestive of a specific outflow graft etiology.

**Figure 2:**
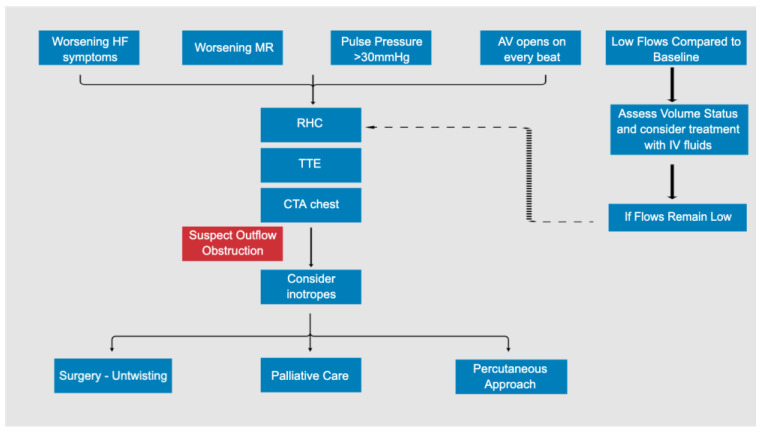
Algorithm for the evaluation of LVAD outflow graft obstruction. AV, aortic valve; CTA, computed tomography with angiography; HF, heart failure; IV, intravenous; MR, mitral regurgitation; RHC, right heart catheterization; TTE, transthoracic echocardiogram.

Our series demonstrates that no single symptom, laboratory result, or echocardiographic parameter is fully reliable for the diagnosis of an outflow graft obstruction. A patient may present with low flow alarms but may be otherwise asymptomatic with stable lab values. Given that some patients are asymptomatic, we are likely underestimating the true prevalence of outflow graft obstructions in the VAD population or possibly diagnosing the condition late in severe cases. The results raise the importance of awareness and urge practitioners to have a higher index of suspicion for outflow graft obstructions when patients present with non-specific symptoms not otherwise explained. CTA is a pivotal noninvasive diagnostic test that can both make the diagnosis as well as elucidate the etiology of an outflow graft obstruction (Figure 2). Decisions on management are best made in a multidisciplinary team of heart failure cardiology, non-invasive cardiology, interventional cardiology, and cardiothoracic surgery teams. The use of fenestrated bend relief may be an important preventative strategy but awaits additional studies to validate its utility [22].

### Limitations

As the number of patients diagnosed with an outflow graft obstruction at our institution is limited, it is difficult to conclude definitive risk factors for patients who develop it. Additionally, without a control group, we are unable to report relative hazards of our outcomes. With rates of obstruction near 3% of the LVAD population, a multi-center or registry-based study may be required to further characterize the patients. An important limitation of our study is that, due to the lack of pathology, we were unable to definitively diagnose outflow tract thrombus, which may affect the interpretation and generalizability of our findings. While the diagnosis of thrombus was presumed based on the clinical scenario and diagnostic findings, it is possible that the accumulation of debris between the bend relief and the graft could have contributed to the obstruction in these patients. Additionally, we highlight that IVUS was not routinely used in our cohort and could have provided additional insights into the pathophysiology of outflow obstruction [23].

## 5. Conclusions

Graft obstructions remain a rare but serious complication. The clinical presentation may be non-specific with low flow alarms occurring in most patients, usually with only mildly elevated LDH levels. Patients with external compression causing stenosis were successfully treated with endovascular stenting. Future multi-center studies are needed in order to estimate the true prevalence, therapy, and outcomes of this entity.

## Figures and Tables

**Table 1:** Clinical Demographics.

Case	Age *	Sex	BMI ^+^	Race	LVAD Type	Status of Therapy	HF Etiology	Antiplatelet, mg Daily ^~^	Anticoagulation ^~^	DM ^#^	HTN ^#^
1	59	M	37.5	Black	HM2	DT	NICM	Aspirin, 325 mg	Warfarin	Y	Y
2	56	M	27.1	Black	HM3	DT	NICM	Aspirin, 81 mg	Warfarin	N	N
3	56	M	31.6	White	HM3	DT	ICM	Aspirin, 81 mg	Warfarin	N	Y
4	75	M	24	White	HM3	DT	NICM	Aspirin 325 mg	Warfarin	N	Y
5	50	M	37.4	White	HM3	DT	NICM	Aspirin, 81 mg	Warfarin	N	Y
6	63	M	48.9	Black	HW	DT	NICM	None	None	Y	Y
7	47	M	44.6	Black	HW	DT	NICM	None	Warfarin	N	Y
8	51	M	34	Black	HW	DT	NICM	None	Warfarin	Y	Y
9	50	M	22.6	Black	HW	BTT	NICM	Aspirin, 325 mg	Warfarin	N	Y
10	43	M	26.4	White	HW	BTT	ICM	Aspirin, 325 mg	Warfarin	Y	Y
11	53	F	35.9	White	HW	BTT	NICM	Aspirin, 325 mg	Warfarin	N	Y

* Age at diagnosis, ^+^ BMI denoted as kg/m^2^, DM diabetes, HF denotes heart failure, HTN hypertension, NICM non-ischemic cardiomyopathy, ICM ischemic cardiomyopathy, BTT bridge to transplant, DT destination therapy. **^~^** reported on home medication list at time of diagnosis, ^#^ defined as problem listed as diagnosis with corresponding medication.

**Table 2:** Presenting Characteristics.

Case	Days to Diagnosis (Years) ^+^	Presenting Symptoms	Cr presentation * (Baseline), mg/dL	LDH Presentation (Baseline), U/L	AST/ALT Presentation (Baseline), U/L	Bilirubin Presentation (Baseline), mg/dL	NT-proBNP Presentation (Baseline), pg/mL
1	2708 (7.4)	Low flow alarms, asymptomatic	2.40 (1.39)	478 (468)	42/57 (54/41)	2.3 (1.6)	426 (1147)
2	238 (0.7)	Low flow alarms, nausea, abdominal pain, dark urine	1.75 (1.62)	383 (175)	19/12 (12/12)	1.3 (0.4)	>35,000 (16,182)
3	118 (0.3)	Low flow alarms, asymptomatic	1.04 (0.67)	340 (202)	30/38 (24/38)	0.5 (0.4)	1104 (1562)
4	596 (1.6)	Low flow alarms, SOB, lightheaded	0.81 (0.74)	129 (179)	20/13 (19/11)	0.4 (0.3)	N/A (N/A)
5	875 (2.4)	Low flow alarms, asymptomatic	0.95 (1.13)	390 (205)	20/17 (22/20)	0.5 (0.4)	787 (384)
6	1422 (3.9)	Low flow alarms, fatigue	3.68 (1.72)	327 (224)	17/3 (17/8)	2.8 (0.7)	N/A (N/A)
7	1476 (4.0)	Low flow alarms, SOB, lightheaded	2.14 (1.58)	266 (244)	26/20 (20/12)	0.4 (0.5)	7116 (2361)
8	1045 (2.9)	Low flow alarms, lightheadedness, syncope	6.75 > (7.0)	178 (191)	12/13 (9/10)	1.4 (1.1)	>35,000 (>35,000)
9	1035 (2.8)	Low flow alarms, asymptomatic	2.33 (1.72)	922 (143)	45/17 (17/14)	1.7 (0.7)	N/A (N/A)
10	143 (0.4)	Low flow alarms, asymptomatic	0.95 (0.83)	171 (198)	40/70 (36/51)	0.6 (0.5)	N/A (N/A)
11	299 (0.82)	No low flow alarms, lightheaded, dizzy	0.95 (1.08)	225 (154)	27/18 (35/27)	0.7 (0.5)	4305 (2937)

^+^ denotes days between LVAD implant and obstruction diagnosis. Parentheses denotes years. * Baseline values were obtained closest to 3 months prior to diagnosis of obstruction, >patient with end-stage renal disease on hemodialysis, LDH denotes lactate dehydrogenase, AST aspartate aminotransferase, ALT alanine aminotransferase, NT-proBNP N-terminal pro b-type natriuretic peptide.

**Table 3:** Diagnosis and Imaging.

Case	Obstruction Type	TTE	CTA	Angiogram, RHC
1	outflow tract stenosis	LV severely dilated (persistent), LVEDD 7.7 cm, AV does not open, severe TR, severe MR (stable); outflow peak 104 cm/s	Proximal 7 cm of the output cannula has a large thrombus causing up to 80% of stenosis of the lumen	Fibrinous debris between the bend relief and the cannula causing severe stenosis. RA 24, PA 44/28 (34), PCWP 24, CO 6.8/3, PVR 1.5, SVR 505
2	inflow and outflow thrombus	LV severely dilated (increased), LVEDD 7.1 cm, AV opens with every beat, moderate TR, severe MR (increased), outflow not well seen	No cannula obstruction, with limited evaluation of the inflow cannula due to streak artifact	LVAD outflow graft angiography with a significant thrombus burden extending from the LVAD motor to the proximal portion of the outflow graft. PA 58/31 (44), CVP 16, PCWP 29, CO/CI 6.28/3.38, SVR 802, PVR 0.24
3	outflow cannula kink	LV normal size (persistent), LVEDD 5.5 cm, AV opens with every beat, trace TR, moderate MR (increased), outflow not well seen	Kink in the distal outflow cannula; patient inflow and outflow of LVAD cannulae	RA 8, PA 42/15 (25), PCWP 10, CO/CI 4.7, 2.4, PVR 3.1, SVR 1384
4	outflow cannula kink	LV mildly dilated (persistent), LVEDD 6.0 cm, AV does not open, mild tricuspid regurgitation (stable), mild–moderate MR (stable), outflow not well seen	Infolding of the proximal portion of the output cannula	N/A
5	outflow tract thrombus	LV severely dilated (persistent), LVEDD 7.1 cm, AV opens with every beat, trace TR, moderate MR (increased), outflow not well seen	Stenosis of proximal LVAD outflow tract, due to thrombus	N/A
6	outflow tract thrombus	LV severely dilated, LVEDD 7.4 cm, AV opens with every beat, moderate–severe TR, moderate MR, outflow not well seen	N/A	Complete occlusion of the LVAD outflow cannula. PA 64/36 (45), CO/CI 7.91/2.77, SVR 506, CVP 20, PCWP N/A
7	outflow tract stenosis	LV severely dilated (persistent), LVEDD 7.4 cm, AV opens with every beat, moderate–severe TR, moderate MR (increased), outflow not well seen	Decreased opacification of the proximal outflow cannula	70% focal stenosis about 1/2 cm from anastamosis identified; PA 64/36 (45), CO/CI 7.91/2.77, SVR 506, CVP 20
8	outflow tract stenosis	LV severely dilated (persistent), LVEDD 8.0 cm, AV opens with every beat, trace TR, moderate MR (persistent), outflow not well seen	No thrombus, moderate stenosis measuring 3 mm in axial dimension	60 mmHg gradient between LVAD outflow graft and the ascending aorta. PA 61/32 (44), RA 14, PCWP 26, CO/CI 4.4/2, PVR 4.11
9	outflow tract thrombus	LV severely dilated (persistent), LVEDD 8.8 cm, AV does not open, mild TR, mild–moderate MR (increased)	N/A	Filling defect in the outflow tract. PA 43/32 (37), RA 14, PCWP, 20, PVR 4.5, CI/CO 3.8/1.9
10	outflow tract thrombus	LV normal size (persistent), LVEDD 4.8 cm, AV opens with every beat, mild TR, mild–moderate MR (increased), outflow not well seen	Nonocclusive outflow cannula thrombus	RA: 12, PA 39/14 (25), PCWP 16, PVR 2.14, CI/CO 4.22/2, SVR 18.45
11	outflow cannula kink	LV moderately dilated (increased), LVEDD 6.1 cm, AV does not open, moderate–severe TR, mild MR (increased); outflow not well seen	Kink in outflow cannula, 50% stenosis	RA: 13, PA: 24/11 (18), CO/CI: 4.1/2.0, SVR 1522, PVR 1.95, PCWP 10

TTE denotes transthoracic echocardiogram, CTA computed tomography angiography, RHC right heart catheterization, LV left ventricle, LVEDD left ventricular end diastolic volume, MR mitral regurgitation, AV aortic valve, TR tricuspid valve, RA right atrial pressure, PA pulmonary artery pressure (mean), PCWP pulmonary capillary wedge pressure, PA pulmonary artery pressure, PVR pulmonary vascular resistance, SVR systemic vascular resistance.

**Table 4:** Intervention and Outcomes.

Case	Obstruction Type and Location	Intervention	Length of Admission, Days	Time to Next Admission, Days	Mortality Status (Days after Diagnosis)
1	outflow tract stenosis	Stent: 11 mm × 79 mm Viabahn VBX	81	49	Alive
2	inflow and outflow thrombus	Supportive care	19	N/A	Deceased (9)
3	outflow cannula kink	Supportive care	11	N/A ^+^	Alive
4	outflow cannula kink	Supportive care	N/A ^	N/A	Alive
5	outflow tract thrombus	LVAD outflow graft revision	21	59	Alive
6	outflow tract thrombus	Supportive care	248	N/A	Deceased (8)
7	outflow tract stenosis	Stent: 11 mm × 39 mm Viabahn VBX	75	168	Alive
8	outflow tract stenosis	Stent: 11 mm × 39 mm Viabahn VBX	8	N/A	Alive
9	outflow tract thrombus	LVAD deactivation with Amplatzer Septal Occluder	2	N/A	Deceased (2)
10	outflow tract thrombus	Transplant	68	947	Alive
11	outflow cannula kink	Increase INR goal 2.5–3.0	6	47	Alive

Patient mortality status at July 2020. ^+^ Patient has not been readmitted as of September 2020. ^ Patient was diagnosed in the outpatient setting.

## Data Availability

The data that support the findings are available on request from the first author, C.J.P.
